# Terrapino: a mobile application for Alzheimer’s risk assessment and cognitive health promotion

**DOI:** 10.3389/fdgth.2025.1719645

**Published:** 2025-12-15

**Authors:** Ross Andel, Katerina Sheardova, Jan Pavlik, Martin Vališ, Jana Amlerova, Jakub Hort

**Affiliations:** 1 Edson College of Nursing and Health Innovation, Arizona State University, Phoenix, AZ, United States; 2 Department of Neurology, Second Faculty of Medicine, Charles University and Motol University Hospital, Prague, Czechia; 3 International Clinical Research Center, St. Anne’s University Hospital, Brno, Czechia; 4 Alzheimerchain Foundation, Prague, Czechia; 5 Research Institute for Biomedical Science, Hradec Kralove, Czechia

**Keywords:** mobile health, cognitive health, Alzheimer's disease, dementia prevention, user engagement, digital health, mobile application, human-centered design

## Abstract

**Objective:**

Mobile health technologies offer scalable opportunities to promote public health, including cognitive health, via education, engagement, and personalized health approach. This study describes the features of the Terrapino mobile application and its users to date, and provide initial evaluation of the ARA score.

**Methods:**

Between December 2022 and December 2024, 8,395 users completed the Alzheimer's Risk Assessment survey, a comprehensive questionnaire developed to collect comprehensive, evidence-based information about Alzheimer's disease risk and protective factors including sociodemographics, health and health history information, lifestyle habits, subjective memory complaints and perceived stress. Most (95%) used the original, Czech version, but English and Spanish versions are also available.

**Results:**

Users were 18–103 years old (mean 57.1 ± 14.5 years), with 46.4% aged 60 years or older. Most (72%) were women and nearly half held a college degree. Despite relatively high education, lifestyle and health characteristics resembled general population trends, suggesting broad accessibility and reach. In a random forest machine learning models, hypertension, going for walks, playing sports and exercising, education, depression, memory complaints, meditation, vegetable intake and the use of olive oil emerged as most influential variables predicting the overall Alzheimer's Risk Assessment score, whether estimated for the entire sample or for those aged 60 + years. The models explained upwards of 80% of variance in the risk score.

**Conclusions:**

This initial examination suggests good feasibility to engage large numbers of individuals in cognitive health promotion through a mobile platform. The early data also suggests good validity of the Alzheimer's Risk Assessment score collected within the application. The initial findings support future efforts to test the application's capacity to contribute to efforts to cognitive health promotion which can be tested through longitudinal research in the upcoming years.

## 1 Introduction

Mobile applications have become an integral part of daily life across all age groups and across a wide range of uses. With widespread acceptance of mobile applications and advancements in AI-driven personalized digital experiences, their potential as a health promotion tool is enormous (1–3). Rapid developments in telemedicine are addressing global healthcare challenges, particularly those related to equitable access, long wait times, healthcare navigation, workforce shortages in remote areas, scalability of diagnostics, and financial pressures from emerging medical innovations (4–6). Digital tools are increasingly recognized as scalable and pragmatic options for early prescreening and identification of individuals at risk for neurodegenerative disorders—potentially *before* they are able to or even consider engaging with formal health care systems (7, 8).

The convergence of technological advancements, growing smartphone adoption among older adults, and improvements in application functionality create an opportune environment to tackle global health issues (6). Among these is the rising prevalence of Alzheimer's disease and related disorders (ADRD), which currently affects approximately 7 million individuals in the U.S (9). and 30 + million worldwide, figures likely to double by 2050. Notably, 40%–45% of ADRD risk is attributed to modifiable factors (10, 11), highlighting opportunities to delay disease onset and reduce overall prevalence. However, reaching at-risk individuals remains challenging.

Mobile applications provide a novel approach to bridging this gap by delivering up-to-date, potentially personalized health information, health screening, and unprecedented access to medical advice and care. Their key advantages include low cost, minimal risk, and broad accessibility across demographic and geographic boundaries. Finally, mobile applications present an opportunity to collect large amounts of data, both cross-sectionally and longitudinally. These data can serve as a source for an exceptionally wide spectrum of studies upstream and provide outcomes that can inform targeted interventions and health programs powered by AI-driven analytics downstream, at relatively low cost and high efficiency.

There has been steady progress in terms incorporating mobile interventions to cognitive health promotion. For example, a recent randomized clinical trial—the LETHE trial—a multinational randomized controlled study testing a digitally supported adaptation of the FINGER lifestyle intervention (12), demonstrated strong feasibility and acceptability of a mobile approach to cognitive health promotion. The positive findings from the LETHE trial pave the way for a real-world, population-wide deployment using a publicly available, preferably free mobile application with self-directed content. In this context, new mobile applications such as the Silvia app (13, 14) provide early promise in terms of positive outcomes on self-perceived memory through combined behavioral, cognitive, and lifestyle interventions guided with the use of this app.

Building on this knowledge, we present initial information about a new mobile application Terrapino. The Terrapino application is developed for self-guided, easy and freely accessible use, which should enable the collection high-volume, longitudinal lifestyle and behavioral data which can be used downstream to develop AI-powered risk prediction and personalized intervention models (3). This paradigm of large, app-based data collection aligns with findings from recent reviews and meta-analyses confirming that digital interaction can produce small to moderate improvements in cognitive function, particularly when sustained over time (15–17). The application builds on existing knowledge regarding risk and protective factors for ADRD and includes a comprehensive, 50 + item survey that collects information about presumed ADRD risk including sociodemographic, health, and lifestyle information and generates the Alzheimer's Risk Assessment (ARA) score.

Building on previous research on the integration of mHealth into cognitive health promotion and research into risk and protective factors for ADRD in general, this paper describes the development, features, and initial user characteristics of the Terrapino app and test the ability of the individual items from the ARA survey to represent the overall ARA score. We hypothesized that the initial data for this digital cohort will demonstrate wide reach beyond users at high risk of ADRD, with users representing population diversity in terms of sociodemographic, health, and lifestyle distribution. We also expected to observe that the total ARA score will be well-represented by the individual items, showing robust properties in terms of analytical accuracy and generalizability, with common ADRD risk factors emerging as the strongest correlates of the overall ARA score for the entire cohort as well as for the targeted cohort of users aged 60 + years.

## 2 Methods

### 2.1 Participants

This study is a baseline assessment of a prospective digital cohort of users of a mobile health app Terrapino (https://terrapino.com/) who completed the Alzheimer's Risk Assessment (ARA) survey that is embedded within the application any time between December 24, 2022 (application launch date) and December 31, 2024. Unlike small-scale volunteer-based or clinical samples or large-scale studies using probability sampling and panel designs, the Terrapino dataset reflects an open, user-initiated, real-world (ecological) sample of app registrants. Users are prompted to complete the ARA survey at first sign-in and are sent regular (currently monthly) reminders if they do not complete the survey. They also receive a reminder to complete the ARA survey again after every 6 months.

For this initial assessment of user enrollment, we observed that a total of 20,952 users downloaded the application and created user accounts, of whom 8,395 (40%) completed the ARA survey. The remaining 12,557 either did not complete the ARA survey within the period of observation for this study or not at all.

### 2.2 Application development and design approach

**Figure 1:**
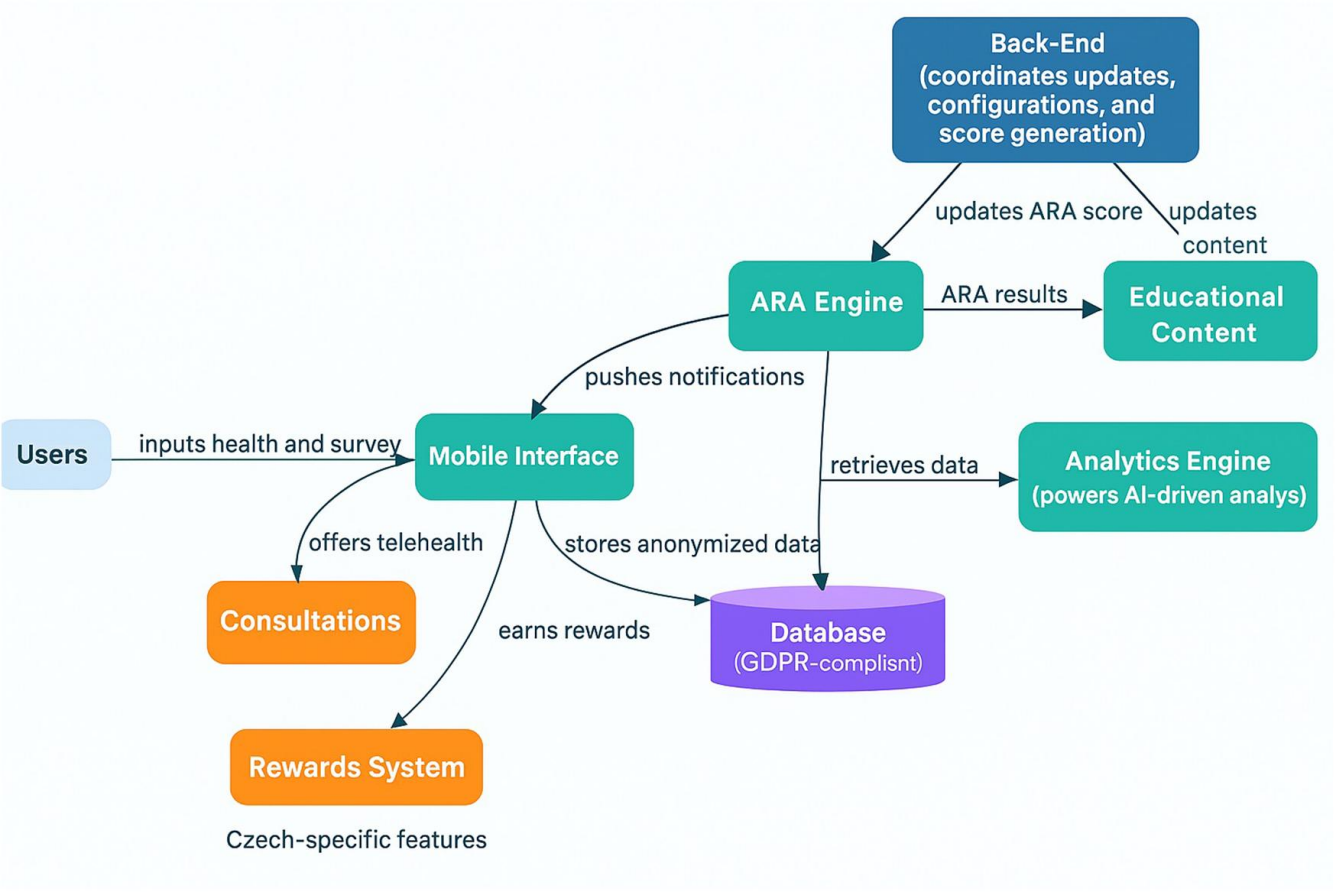
Terrapino Mobile application architecture**.** This figure presents an overview of Terrapino's core architecture. Users interact with the *Mobile Interface* (teal), which collects health and lifestyle inputs and stores anonymized data in a GDPR-compliant *Database*. The *Back-End* coordinates configuration updates, system settings, and ARA score generation, sending updates to the *ARA Engine*. The ARA Engine (Alzheimer's risk assessment engine) processes user data—informed by the *Analytics Engine's* AI-driven insights—to compute an *ARA score*. It delivers results to *Educational Content* modules, which provide personalized feedback to users via the app. Two Czech-specific modules (orange) extend the platform: a *Rewards System* (incentivizing healthy behavior) and *Consultations* (offering telehealth expert consultations). These Czech-specific features are visually distinguished in the diagram, as indicated by the label underneath.

The Terrapino application, available on Apple Store and Google Play, was designed to support cognitive health across individuals of all backgrounds and ages by focusing on three core areas: cognitive training, physical activity, and maintaining a healthy lifestyle. It was developed using a human-centered design framework (18) that emphasized user feedback, intuitive usability, and engagement, particularly for older adults and individuals at risk for cognitive decline. Initial development involved informal consultations with Alzheimer's disease and related disorders (ADRD) specialists, software developers, and clinicians, followed by formal input from the scientific collaborators and healthcare professionals. The app is available for free download on both Apple Store and Google Play. As of December 2024, the app was available in Czech, English, Italian, and Spanish Figure 1).

### 2.3 Application architecture and features

**Figure 2:**
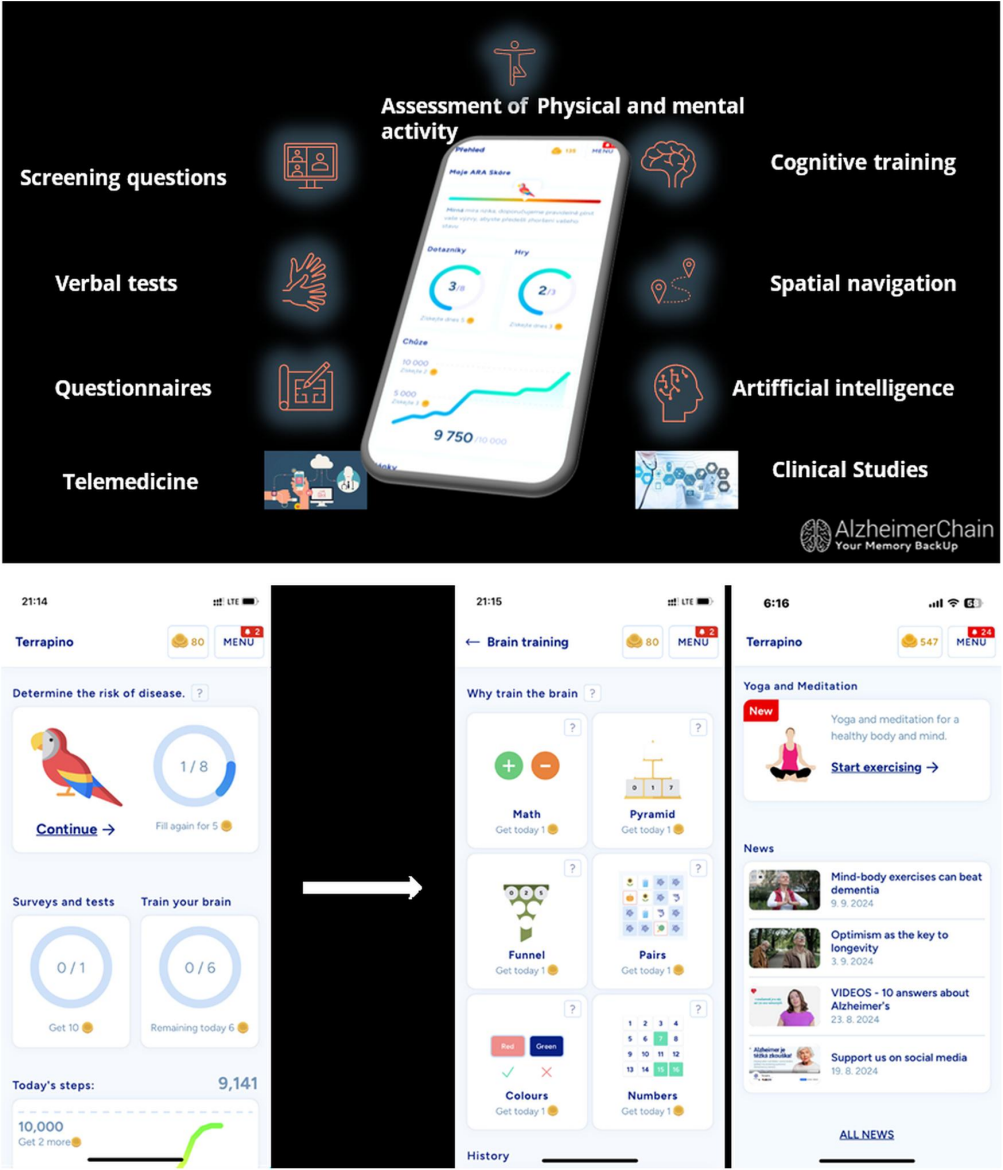
Application features.

The app is structured around a modular design and, in version 1.4.5 (released in December 2024), includes these core features: *A landing page with an Alzheimer's Risk Assessment (ARA) score*, *brain training games*, *physical activity tracking* (steps), *yoga and meditation exercises*, *educational content*, *surveys and tests*, and *general health information* (see Figure 2). Additional sections provide users with contact options, app notifications, FAQs, and personal account settings. For users who utilize the original, Czech language version of the Terrapino app, there are three additional modules: A rewards module, clinical study enrollment options, and a monitored ask-a-specialist feature. The rewards module incentivizes the application users by allowing them to earn coins for activity (e.g., 5 points for completing a puzzle, 10 points for reaching a daily threshold for the number of steps, etc.), which can be redeemed at participating partners (e.g., 20 coins = free coffee; 30 coins = theater discount; and other rewards and discounts at participating businesses, pharmacies, and cultural entities). These additional modules are currently under development in the English and Spanish versions of the app.

The app's content, including risk assessment algorithms and educational materials, is regularly reviewed and updated by the Advisory Board based on current research. The ARA score was developed over time through regular Advisory Board meetings, with the members of the Advisory Board using their extensive clinical and research-based experience to form the structure of the ARA survey as well as weightings of each included factor. Of note is that the decisions were influenced heavily by current evidence regarding Alzheimer's risk and protective factors, which were summarized in the 2020 Lancet Commission report on dementia prevention, intervention, and care (10).

The ARA Survey consists of 8 sections collecting information about (1) Sociodemographics, (2) Social Environment, (3) Health and Medical History, (4) Physical Activity, (5) Intellectual Activity, (6) Nutrition (see Tables 1, 2), (7) Stress, and (8) Memory (see Appendix A for Perceived Stress and Subjective Memory Complaints scales). The ARA scores were weighted by consensus meeting of the Advisory Board according to their relative importance for overall risk of ADRD. The ARA Survey and the weighting scheme are continuously updated in response to new discoveries and publications. The ARA Survey version presented in this study weights the sections as follows: *Health and Medical Diagnosis*—42%; *Nutrition*—11%; *Physical Activity*—9.6%; *Intellectual Activity*—8.9%; *Social Environment*—8.5%; *Memory*—8.5%; *Sociodemographic*—6.5%; *Stress*—5.1%. The resulting possible ARA scores range from −79 (lowest ADRD risk) to +158 (highest ADRD risk). Educational modules offer interactive information on maintaining brain health and reducing Alzheimer's disease risk.

**Table 1:** User characteristics of the entire sample (*n* = 8,395) and those 60 + years old only (*n* = 3,899).

Variable	Entire sample		60 years and older	
	M or *n*	SD or %	M or *n*	SD or %
Sociodemographics				
Age	57.1	14.5	69.7	6.5
Female	6,009	71.6	2,760	70.8
Education level				
Elementary or less	200	2.4	87	2.2
Trade school	876	10.4	444	11.4
High school	3,464	41.3	1,712	43.9
College or more	3,855	45.9	1,656	42.5
Social environment				
Living alone				
With someone	6,133	73.1	2,681	68.8
Alone but with contacts	1,101	13.1	524	13.4
Alone	1,161	13.8	694	17.8
Health and medical history				
Body mass index	26.7	5.1	27.0	4.6
ADRD	140	1.7	110	2.8
Cancer	396	4.7	279	7.2
Hypertension	2,622	31.2	1,799	46.1
Hypotension	881	10.5	363	9.3
Diabetes mellitus	593	7.1	446	11.4
Ischemic heart disease	283	3.4	246	6.3
Stroke	146	1.7	100	2.6
Heart attack	124	1.5	99	2.5
Cholesterolemia	2,899	34.5	1,990	51.0
Depression	1,235	14.7	565	14.5
Hearing problems	2,416	28.8	1,645	42.2
Concussion				
One	1,390	16.6	591	15.2
More than one	515	6.1	198	5.1
Serious head injury	735	8.8	366	9.4
Relatives with dementia				
One	2,619	31.2	1,229	31.5
More than one	995	11.8	394	10.1

M, mean; SD, standard deviation; BMI, body mass index.

**Table 2:** Lifestyle information for the entire sample (*n* = 8,395) and those 60 + years old (*n* = 3,899).

Variable	Entire sample		60 years and older	
	M or *n*	SD or %	M or *n*	SD or %
Health and medical history (cont.)				
*Over the past year…..*				
Vaccinated for COVID				
Once	301	3.6	88	2.3
More than once	7,159	85.3	3,520	90.3
Vaccinated for the flu	3,056	36.4	1,857	47.6
Vaccinated for lime disease	3,555	42.4	1,767	45.3
Vaccinated for pneumococcus	1,307	15.6	805	20.7
Took antibiotics	3,201	38.1	1,459	37.4
Had COVID/what symptoms				
Mild	2,489	29.7	1,052	27.0
Severe (ICU/oxygen)	22	0.2	14	0.4
Sleep 7–8/day	5,463	65.1	2,496	64.0
Snoring while sleeping				
Mild	4,588	54.7	2,323	59.6
Loud/sleep apnea	850	10.1	419	10.8
Physical activity				
Go for long walks				
Occasionally	5,417	64.5	2,424	62.2
Frequently	1,243	14.8	657	16.9
Participate in organized sports				
Occasionally	4,034	48.1	1,551	39.8
Frequently	295	3.5	121	3.1
Engage in exercise				
Occasionally	4,396	52.4	1,843	47.3
Frequently	855	10.2	572	14.7
Meditate yoga Tai Chi etc.				
Occasionally	2,109	25.1	811	20.8
Frequently	730	8.7	309	7.9
Intellectual activity				
Special memory training	565	6.7	400	10.3
Play board games/social games	1,122	13.4	593	15.2
Sudoku	1,711	20.4	1,250	32.1
Solve crossword puzzles	2,184	26.0	1,593	40.9
Read books				
Occasionally	4,312	51.4	1,876	48.1
Daily	1,826	21.8	1,100	28.2
Nutrition				
How many meals/day mean (SD)				
2–3	4,214	50.2	2,104	54.0
4–5	3,117	37.1	1,396	35.8
More	1,064	12.7	399	10.2
Restrict eating times				
Occasionally	2,092	24.9	890	22.8
Frequently	533	6.4	229	5.9
Consume sweetened soft drinks				
Occasionally	3,060	36.5	1,074	27.6
Frequently	433	5.2	124	3.2
Eat highly processed foods				
Occasionally	4,752	56.6	2,097	53.8
Frequently	934	11.1	270	6.9
Eat nuts				
Occasionally	4,770	56.8	2,071	53.1
Frequently	3,307	39.4	2,842	72.9
Drink unsweetened coffee/black tea				
Occasionally	1,557	18.6	704	18.1
Frequently	6,027	71.8	2,842	72.9
Use olive oil				
Occasionally	4,285	51	2,041	52.4
Frequently	3,194	38.1	1,340	34.4
Eat fish				
Occasionally	6,111	72.8	2,868	73.6
Frequently	1,502	17.9	729	18.7
Eat meat products				
Regular consumption	6,514	77.6	505	13.0
A lot	649	7.73	3,354	86.0
Eat vegetables				
Occasionally	2,822	33.6	1,261	32.3
Frequently	5,538	65.9	2,627	67.4
Eat any type of berries				
Occasionally	6,381	76.0	2,986	76.6
Frequently	1,581	18.8	714	18.3
Supplement with Gingko Biloba				
Occasionally	1,381	16.5	786	20.2
Frequently	354	4.2	247	6.3
Get sun exposure/take vitamin D				
Occasionally	2,463	29.3	972	24.9
Frequently	5,591	66.6	2,802	71.9
Drink alcohol				
Occasionally	5,582	66.5	2,633	67.5
Frequently	1,231	14.7	488	12.5
Smoke	984	11.7	341	8.8
Stress				
Perceived stress scale	14.0	6.3	12.7	5.7
Memory				
Subjective memory complaints	1.7	1.7	1.8	1.7

M, mean; SD, standard deviation.

### 2.4 Ethical approval

Only users aged 18 + years who agreed to use the anonymized data for research purposes at the initial user account activation were considered. No personal identifiers were collected, and all data were stored in compliance with the General Data Protection Regulation (GDPR) (19). The application meets all security and confidentiality requirements as a certified medical device under the European Union's Medical Device Regulation—Risk class I; Basic UDI: 859421939TERRAPINODV)**,** which includes standardized and general terms and conditions of use agreed by users upon initial login. Arizona State University Internal Review Board (IRB) approved processing and analyses of the app data.

### 2.5 Data collection and measures

The data presented here are all part of the ARA score, which was designed based on current knowledge of risk and protective factors for Alzheimer's disease, with decision heavily influenced by the two recent Lancet Commission reports on dementia prevention, intervention, and care (10). Based on this knowledge, app uses integrated questionnaires to collect comprehensive demographic, lifestyle, and health information that is processed through an algorithm to produce an individual ARA score that is shared with the user. Demographics include age, sex, education, and living arrangement. Health information includes diagnoses of hypertension, diabetes, cardiovascular disease, depression, and cognitive impairment (see Table 1 for demographic and health information). Lifestyle factors include dietary habits, exercise routines, cognitive activities (e.g., puzzle use), sleep quality and duration, and wellness practices such as yoga or meditation (see Table 2). The Perceived Stress Scale (PSS-10) (20) was used to evaluate perceived stress, while a custom list of eight binary questions assessed subjective memory complaints (see Appendix A). The ARA score is generated only upon the completion of all components of the ARA score.

In addition to the information that is part of the ARA score, step count and exercise frequency are collected through direct user input and through passive tracking (i.e., step count). Engagement with cognitive and meditation modules is automatically recorded for number of clicks and lengths for which the module was open. The app is continuously evolving and new app functionalities are being added. For example, during the second half of 2024, the app was enriched with step count data, which was available for 1,891 users of the 8,395 users with complete data as of December 31, 2024. Also, a short test of semantic and phonological fluency (21) was adopted for use within app and is being beta tested.

### 2.6 Analysis

Means and standard deviations or n's and percentages are presented for the ARA Survey variables. We used machine learning, applying the random forest algorithm to assess which items from the main application survey contributed most strongly to the total ARA score. To do so, we used the R package *ranger* (22), a newer and more efficient implementation of the R package *randomforest* which is particularly suitable for high-dimensional data like ours. The data were randomly divided into training (70%) and testing (30%) subsets, with the random forest algorithm allowed to train on the 70% training set using 5-fold cross-validation and 800 trees. Model performance was then evaluated on the testing set by root-mean-square error (RMSE), mean absolute error (MAE), and coefficient of determination (R²). The Shapley Additive Explanations (SHAP) (23) and percent of ARA score variance explained were outputted for the top 10 contributors to the ARA score. Finally, we estimated the same algorithm with users aged 60 + years only. Users with ADRD were excluded from these analyses.

## 3 Results

### 3.1 Descriptive data overview

A total of 8,395 Terrapino app users provided complete data as of December 31, 2024. The user characteristics overall and for the targeted age range of 60 + years are presented in Tables 1, 2. Users ranged in age from 18 to 103 years (mean age 57.1, SD 14.5), with 3,899 (46.4%) of the 8,395 being in the targeted age range of 60 + years of age. The majority of participants (∼71%) were women and most >85% had at least high school education, of whom >40% were college educated. About a quarter lived alone. Characteristics were similar for the entire sample and those 60 + years old except for higher occurrence of hypertension, cholesterolemia and hearing problems in the 60 + subgroup of Terrapino application users. Health and lifestyle factors in Table 2 showed a relatively high frequency of vaccinations and good overall engagement in physical activity. Most drank alcohol at least occasionally and only a small proportion were smokers.

### 3.2 Random forest regression

After excluding participants diagnosed with ADRD, the analytic dataset comprised individuals with a normally distributed continuous outcome (*ARA Score*) ranging from −46 to 79 (mean = −9.5, median = −10; IQR = −19 to −2) and 8,255 observations. Continuous predictors included age, BMI, perceived stress and memory; all other predictors were categorical and treated as factors.

The results of the random forest regression to isolate the main contributors to the ARA score demonstrated strong predictive performance (RMSE = 4.8, MAE = 3.68, R² = 0.87), indicating good accuracy and generalization, with 87% of the variance in the ARA score account for. Variable contributions were assessed using mean absolute Shapley Additive Explanations (MA-SHAP). The ten most influential variables in terms of explaining variance in the overall ARA score are shown in the top portion of Table 3.

**Table 3:** Top Ten predictors of the overall ARA score ranked by mean absolute shapley additive exPlanations (MA-SHAP) value.

Predictor	MA-SHAP	Percentage
Entire sample		
Hypertension	2.8	12.5
Walks	2.6	11.4
Playing sports	2.4	10.8
Education	2.1	9.4
Depression	1.6	7.1
Subjective memory complaints	1.2	5.4
Exercise	1.1	4.9
Meditation	0.7	3.3
Eating vegetables	0.7	3.1
Using olive oil	0.7	2.9
Those 60 + years old		
Hypertension	2.9	14.0
Walks	2.7	13.1
Playing sports	2.2	10.9
Education	2.1	6.5
Depression	1.2	6.0
Exercise	1.0	4.8
Diabetes	0.9	4.5
Subjective memory complaints	0.9	4.4
Meditation	0.6	2.7
Using olive oil	0.5	2.7

When this sample was reduced to only those users aged 60 + years, the analytic dataset still included a roughly normally distributed continuous outcome (*ARA Score*) ranging from −46 to 79 (mean = −7.45, median = −8; IQR = −16 to 1) and 3,789 observations. Using the same procedure as with the entire sample, the model demonstrated strong predictive performance (RMSE = 5.41, MAE = 4.06, R² = 0.84), again indicating good accuracy and generalization, with 84% of the total variance in the ARA score explained by the included variables. The second portion of Table 3 shows the two most influential variables in this model.

## 4 Discussion

*Terrapino* is a mobile application designed to promote cognitive health and reduce risk for Alzheimer's disease and related disorders (ADRD) through a scalable, technology-supported platform. Our objective was to present initial information regarding the development and features of the Terrapino mobile application, describe the characteristics of the first users who completed the comprehensive the Alzheimer's Risk Assessment (ARA) survey within the application, and test the ability of the individual items from the ARA survey to represent the overall ARA score. In the two years since its launch, *Terrapino* has reached over 20,000 users, with 8,395 individuals providing complete data on the comprehensive Alzheimer's Risk Assessment (ARA), which was developed using most recent evidence regarding risk and protective factors for ADRD (10). The app attracted a broad demographic, with uptake across adults of all ages, with those 65 years or older, to whom the application is targeted, comprising only 35% of the 8,395 total active users. This broad adoption suggests the feasibility of engaging diverse age groups, not just those at most risk or already experience incipient signs of cognitive impairment, with digital tools aimed at cognitive health promotion.

While only about one-third of users were aged 65 and older—the conventional target demographic for ADRD prevention—this age group was substantially represented, and the unexpectedly high engagement among middle-aged adults (40+) reinforces growing evidence of smartphone adoption and digital readiness across the adult lifespan (6). This finding supports the feasibility of mobile health (mHealth) interventions for brain health not only in older adults, but also earlier in adulthood, when some preventive measures may be most beneficial (11). In addition, the enrollment of a relatively large number of younger individuals could indicate a combination of (a) general, growing propensity to pay attention to cognitive health earlier in adulthood and (b) an app-specific tendency for young people, potentially relatives, to enroll in support of older application users.

The user base demonstrated meaningful engagement in health-related behaviors, including frequent physical activity, cognitive stimulation (e.g., reading, puzzles), and healthy dietary patterns. These behaviors are consistent with the app's core design and suggest that Terrapino may successfully encourage lifestyle habits associated with lower ADRD risk, although they do not immediately inform about the app's potential to modify such behaviors.

The initial observations presented in this study map well on knowledge regarding risk and protective factors for ADRD (11). Specifically, results of the random forest regression conducted to evaluate main contributing factors to the overall ARA score among the ADRD-free users yielded hypertension as the most influence factor, followed by going for walks, engaging in sports, education, depression, subjective memory complaints, exercise, mediation, eating vegetables, and the use of olive oil. With small deviations, the same variables contributed most to the ARA score in users 60 years of age and older. This initial information suggests good psychometric properties of the ARA score, although longitudinal follow-up and analyses are needed to properly evaluate the properties. These analyses are planned as data matures over the years and decades, providing opportunities for refinement of the ARA score and gathering of new knowledge about the utility of the scale and this digital platform to assess the risk of ADRD and its contribution to health promotion and ADRD prevention.

Also planned are analyses of individual items, all collected from tens of thousands of users, to test hypotheses regarding gender differences, the contribution of living alone to health outcomes and adherence to healthy lifestyle habits, correlates and predictors of subjective memory complaints and perceived stress to name a few. Terrapino's modular structure and adaptive content may be especially well-suited to test a wide spectrum of hypotheses over time.

Terrapino's design attempts to address several known limitations of traditional health education efforts—namely, cost, scalability, personalization, and continuity. The inclusion of features such as the ARA score, virtual expert consultations, gamification and a reward-based engagement system (in the original Czech version) enhances both interactivity and sustained use. Importantly, this model aligns with the Technology Acceptance Model (TAM), which emphasizes perceived usefulness and ease of use as key drivers of digital adoption (24), and is further grounded in human-centered design (HCD) principles that prioritize empathy, feedback, and responsiveness to user needs (18, 25), which can guide efforts in population-wide recruitment and long-term user retention and engagement.

The findings also complement recent evidence from the LETHE trial testing the FINGER lifestyle intervention, which demonstrated strong feasibility and acceptability of digital cognitive health strategies among older adults (12). Like LETHE, *Terrapino* leverages both in-app behavior tracking and wearable-integrated features to support personalized health monitoring. Unlike LETHE, *Terrapino* is deployed in a real-world, self-directed context and is widely available, reinforcing its potential for population-level implementation. The initial observations from Terrapino hold promise in terms of downstream expansion of findings demonstrating the efficacy of mobile-based approach to improvement health outcomes including cognitive function (13, 14). Rather than a restrictive trial approach, Terrapino was designed to collect high-volume, longitudinal data, creating an opportunity for future development of AI-powered risk prediction and personalized intervention models (3).

Spitzer's hypothesis of the digital dementia (26, 27) suggests that many years of technology exposure worsens cognitive abilities, whereas the opposing hypothesis claims that digital exposures may increase technological reserve, wherein digital technologies promote behavioral patterns that preserve cognition (28). Although concerns persist that digital technologies may contribute to cognitive decline, recent review analyzing 136 publications (29) suggests that older adults who use smartphones and tablets may actually experience slower rates of cognitive decline. Still, it remains unclear whether this reflects a causal effect or a selection bias in favor of more cognitively intact users. Terrapino and other applications capable of collecting large volumes of data provide an opportunity to generate answers to these questions.

Terrapinós modular structure and adaptability reflect core features identified as critical to effective interventions, namely personalization, gamification, and integration of cognitive and lifestyle data (30). This and similar scalable digital tools are urgently needed to meet the challenges posed by the projected doubling of global dementia prevalence by 2050 and the substantial portion of risk that remains modifiable (9, 11).

Terrapino's self-directed, real-world model has so far attracted a relatively large and demographically diverse user base, while the app's multilingual availability and iterative, science-driven updates align it with best practices in digital public health. Moreover, *Terrapino* offers a flexible infrastructure capable of expanding or adapting modules based on evolving user needs and scientific evidence.

Several limitations must be considered. First, because app usage is voluntary and initial engagement is not incentivized, user makeup is prone to a self-selection bias towards self-motivated or already health-conscious individuals. Future research should study strategies to recruit and retain a wide spectrum of users with respect to the desire to engage in health promoting behaviors, such as the inclusion of incentives. Second, while the app is multilingual, most users to date engaged with the Czech-language version, limiting immediate generalizability across cultural contexts. Third, reliance on self-reported data introduces potential inaccuracies due to recall bias or socially desirable responses. Four, the ARA score which the application generates based on comprehensive self-reported information across its 8 sections has not yet been validated. Finally, as this report is based on cross-sectional data, no causal inferences can yet be drawn regarding the app's impact on behavior or cognition. Nonetheless, the app was designed to support longitudinal use, and future analyses will be able to assess changes in health behaviors and cognition over time. The approach to user retention via incentivizing app use should facilitate longitudinal data capture.

There are several directions for future exploration. Ongoing development of applications like *Terrapino* includes expanding personalization, AI-based tailoring, and support for broader international rollout. Longitudinal data will allow researchers to validate application-based data as a predictive tool and examine behavioral change over time. As evidence continues to mount for the value of early, multimodal, and modifiable-risk interventions in ADRD prevention, mobile applications are well positioned to contribute as a cost-effective, scalable solution that integrates health promotion with large-scale digital data capture.

In conclusion, *Terrapino* represents the next generation of mobile tools that combine user-centered design, real-world accessibility, and scientific rigor to promote cognitive health population-wide. It appears quite successful in terms of recruiting users across ages, health, and lifestyle habits, although education appears to play an important role in engagement with the application. Its built-in capabilities for ongoing risk assessment, user interaction, and behavioral support highlights that this and similar mHealth applications can play a critical role in population-wide dementia prevention in the digital age. The initial uptake indicates strong demand for accessible digital solutions supporting cognitive health. With global dementia rates expected to continue to rise, scalable digital interventions like *Terrapino* may be key to reducing risk and enhancing cognitive resilience in aging societies.

## Data Availability

The raw data supporting the conclusions of this article will be made available by the authors, without undue reservation.

## Appendix A. Items to assess stress management and memory/cognitive complaints

**Perceived Stress Scale** (20).

How often have you gotten angry over the past month because of something that happened unexpectedly?

How often have you felt over the past month that you are not able to control important things in your life?

How often have you felt nervousness and stress over the past month?

How often have you felt confident in your ability to handle your personal problems over the past month?

How often have you felt over the past month that things are developing according to your plans?

How often have you found over the past month that you cannot cope with all the responsibilities you had to do?

How often were you able to control your irritability over the past month?

How often have you felt over the last month that you were on top of things?

How frequently did you get so angry something happened last month that you couldn't control yourself?

How often did you feel last month that troubles were piling up so much that they could not be overcome?

**Subjective memory complaints (yes/no)**

Over the past 6 months, have you felt that you remember events worse, or have others told you, “I already told you that”?

Have you forgotten an important appointment in the last 6 months?

Have you lost your things more often or for longer periods in the last 6 months than usual?

Have you had difficulty orienting yourself in space or not recognizing places you've been told you

Have you completely forgotten about an experienced event, even when your loved ones talked about it or when you saw it again in photographs?

Have you felt that during a conversation you were searching for words (excluding proper names) and had to use different words, or did you find yourself pausing or saying “um” more often than usual?

Have you limited certain activities or asked others for help out of fear of making mistakes? (personal and work-related matters)

Have you noticed that your personality has changed—withdrawing into yourself, reducing contacts with others, or feeling less interested in things or less initiative?
